# ﻿Genus *Hainanphasma* Ho, 2013: taxonomic notes and two new species from Hainan in China (Phasmida, Heteropterygidae)

**DOI:** 10.3897/zookeys.1232.140688

**Published:** 2025-03-25

**Authors:** Yi-Fan Liu, Jun-Jie Gu, Hai-Jian Wang

**Affiliations:** 1 College of Agronomy, Sichuan Agricultural University, Chengdu 611130, China Sichuan Agricultural University Chengdu China

**Keywords:** Datamini, key, Phasmatodea, Stick insects, taxonomy

## Abstract

Two new species of *Hainanphasma* Ho, 2013, *Hainanphasmalongiacuta***sp. nov.** and *H.longidentata***sp. nov.**, are described and illustrated based on specimens of both sexes from Hainan, China. The heads of *H.cristata* Ho, 2013, based on both sexes, and *H.diaoluoshanensis* Ho, 2013, based on females, are redescribed from new materials. The generic diagnosis of *Hainanphasma* is improved, and the distinction from *Orestes* Redtenbacher, 1906 is clarified. The male and egg of *H.diaoluoshanensis* are described and illustrated for the first time. A key to species of *Hainanphasma* is provided.

## ﻿Introduction

The Datamini Rehn & Rehn, 1939 currently consists of five genera distributed in China, with two in Hainan Province: *Hainanphasma* Ho, 2013 and *Planispectrum* Rehn & Rehn, 1939 (Ho 2016; Brock et al. 2024). The genus *Hainanphasma*, which is endemic to Hainan, was described in 2013 and attributed to the tribe Datamini, subfamily Dataminae, of the Heteropterygidae (Ho 2013; Brock et al. 2024). This genus includes two species, characterized by the following characters (Ho 2013). In both sexes, the antennae are slender and thin. In females there is a posteriorly projecting and punctulate occipital crest, and the thorax is parallel-sided, with a faint median carina. In males, the occipital crest is strongly elevated, there is a fin-like lamella on the postero-dorsal carina of the meso- and metafemora, the fourth and fifth abdominal terga have distinctive humps, and the thorax is unarmed but with an indistinct median carina. Nonetheless, differentiating *Hainanphasma* from other genera in Datamini, particularly *Orestes* Redtenbacher, 1906 is challenging based on the existing characters.

Bresseel and Constant (2018) have previously examined this genus, presenting specific examples that highlight challenges with diagnostic characters. Gao and Xie (2022) also noted the limited generic differentiation of this genus. The monophyly of *Orestes* has been confirmed by phylogenetic analyses (Bank et al. 2021), but the phylogenetic relationship between *Orestes* and *Hainanphasma* is currently unclear due to the lack of *Hainanphasma* material.

**Figure 1.**
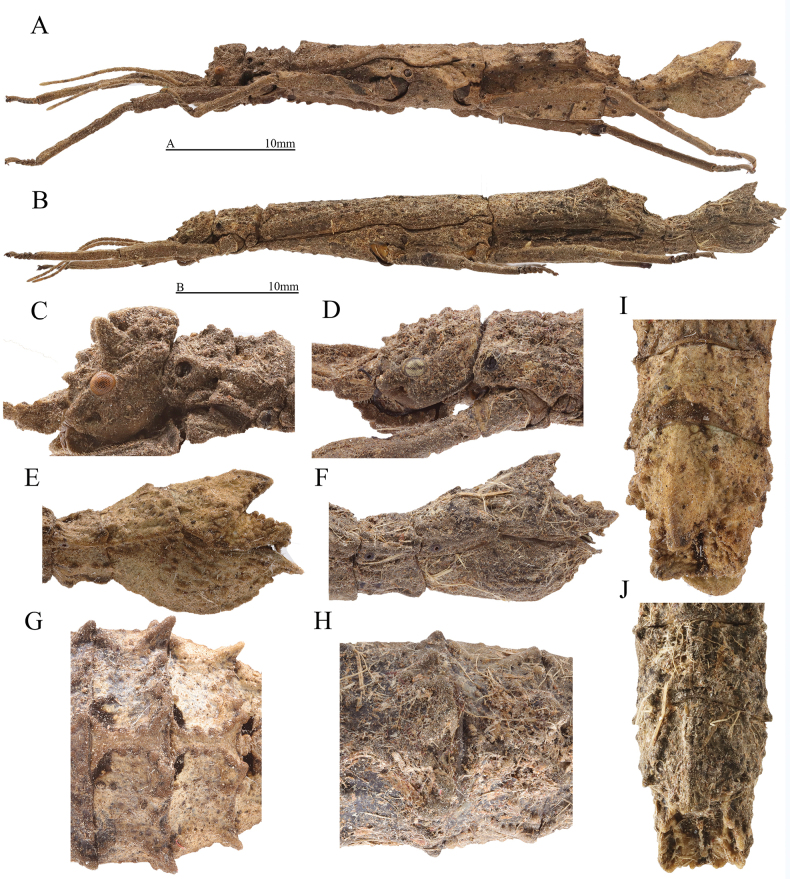
Female of *Orestesguangxiensis* (Bi & Li, 1994) from China, Guangxi, Bobai, 15 Jul. 2022, Yunxiang Zhang leg.; female of *Orestesmouhotii* (Bates, 1865) from Thailand, Chiang Rai, Ban Nanglae Nai, 11 May. 2024, Jie Su leg. **A, B** lateral view of habitus **A***O.guangxiensis* (Bi & Li, 1994) **B***O.mouhotii* (Bates, 1865) **C, D** lateral view of head **C***O.guangxiensis* (Bi & Li, 1994) **D***O.mouhotii* (Bates, 1865) **E, F** lateral view of terminalia **E***O.guangxiensis* (Bi & Li, 1994) **F***O.mouhotii* (Bates, 1865) **G, H** dorsal view of fourth to fifth abdominal terga **G***O.guangxiensis* (Bi & Li, 1994) **H***O.mouhotii* (Bates, 1865) **I, J** dorsal view of terminalia **I***O.guangxiensis* (Bi & Li, 1994) **J***O.mouhotii* (Bates, 1865). Scale bars: 10 mm (**A, B**); **C–J** not to scale.

**Figure 2.**
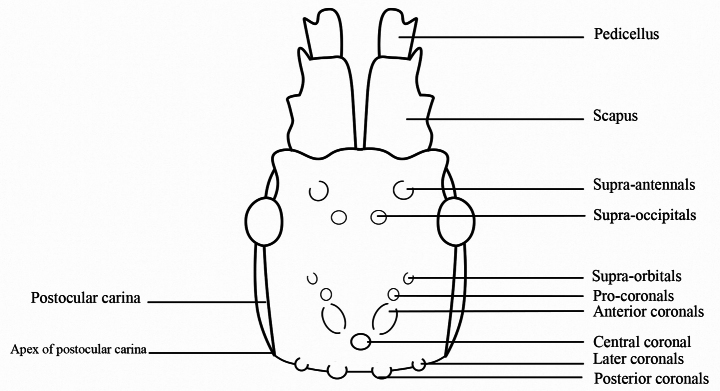
*Hainanphasma* Ho, 2013, nomenclature of cephalic armature.

In this paper, we describe two new species and new materials of *H.diaoluoshanensis* Ho, 2013. These findings refine our understanding and clarify its generic status. The paper provides images of female of *O.guangxiensis* (Bi & Li, 1994) collected from Guangxi province, China and *O.mouhotii* (Bates, 1865) collected in Chiang Rai, Thailand to ensure the representative and stable characters used for comparison with *Hainanphasma* in this paper (Fig. 1). *Orestesmouhotii* is the type species of *Orestes*, and *O.guangxiensis* is distributed in Guangxi and Guangdong in China, which are the closest provinces to Hainan. During collection in different areas of Hainan, two new species, *H.longiacuta* sp. nov., *H.longidentata* sp. nov., and the male and egg of *H.diaoluoshanensis* Ho, 2013 were discovered. The arrangement of cephalic armature is figured (Fig. 2), showing similarities to *Microrestes* Bresseel & Constant, 2020, but the cephalic armature differs in having supra-orbitals absent or indistinct and a central coronal present. The heads of *H.cristata* Ho, 2013, based on both sexes, and *H.diaoluoshanensis* Ho, 2013, based on females, are redescribed. A key to species of *Hainanphasma* is provided.

## ﻿Materials and methods

Specimens were preserved in ethanol in the field, pinned, and dry-preserved in the laboratory. The holotype and some paratypes are deposited at Department of Plant Protection of Sichuan Agricultural University (SICAU), Chengdu, China.

The habitus picture of the specimens were photographed with a Canon EOS550D camera equipped with a Canon EF 100 mm f/2.8L IS USM and a Canon SPEEDLITE 470EX-AI flash (Canon China, Beijing, China). The other images were taken with a SZX16 microscope system and cell Sens Dimension 3.2 software (Olympus, Tokyo, Japan) or a Leica M205A digital imaging system (Leica, Wetzlar, Germany) in the State Key Laboratory of Crop Gene Exploration and Utilization in Southwest China, Sichuan Agricultural University. Measurements were taken under a stereomicroscope with vernier calipers or eyepiece micrometer.

The morphological terms were followed Bragg (2001), Zompro (2004), and Bradler (2009). The nomenclature of the cephalic armature were followed Bresseel and Constant (2020) and Hennemann et al. (2016).

## ﻿Results

### ﻿Genus Hainanphasma

Taxon classificationAnimaliaPhasmidaHeteropterygidae

Ho, 2013

6BA0E909-7EA8-5342-BC30-E90AC09124E0

#### Type species.

*Hainanphasmacristata* Ho, 2013, by original designation.

#### Included species.

*H.cristata* Ho, 2013, *H.diaoluoshanensis* Ho, 2013, *H.longiacuta* sp. nov., *H.longidentata* sp. nov.

#### Revised diagnosis.

The genus *Hainanphasma* differs from other genera of the Datamini Rehn & Rehn, 1939 by the following combination of characters:

The arrangement of cephalic armature (Fig. 2): supra-occipitals present as blunt spines or small elevations, not split into anterior and posterior supra-occipitals; supra-orbitals absent or indistinct; central coronal present.

In female, body cylindrical; third to fourth abdominal terga slightly increasing in width; postero-dorsal carina of meso- and metafemora with fin-like lamellae or teeth; antero-dorsal and ventral carinae of meso- and metafemora with small teeth of irregular sizes; fifth abdominal tergum with distinct tubercles and lamellae posteriorly, higher than fourth abdominal tergum; ninth abdominal tergum with distinct posteromedian crest, from slightly shorter to distinctly longer than anal segment.

In male, second abdominal tergum rectangle, longer than wide; dorsal and ventral carinae of meso- and metafemora with fin-like or semi-circle teeth of irregular sizes; posterior margin of fifth abdominal tergum with a pair of humps.

Eggs capsule brown to darker brown, bearing very sparse setae; micropylar plate with margin elevated and broadened; operculum subcircular, elevated centrally; ventral margin oblique in lateral view.

The most closely related genus is *Orestes*. But in *Orestes*, supra-occipitals split into anterior and posterior supra-occipitals; supra-orbitals distinct, present as blunt spines or lamellae (Fig. 1C, D). In female, body robust with widest part at the posterior margin of fourth abdominal tergum or fourth and fifth terga parallel-sided; fourth and fifth abdominal terga with tubercles posteriorly, anterior one larger and higher (Fig. 1G, H); ninth abdominal tergum with small posteromedian crest, not reaching posterior margin of anal segment, shorter than anal segment (Fig. 1E, F, I, J). Eggs can be distinguished by capsule and operculum covered with long and dense setae; operculum shape variable elongated to circular, without distinct elevations.

#### Distribution.

China, Hainan province.

### ﻿Key to species of *Hainanphasma*

Figs 3, 4

Females

**Table:** 

1	Posteromedian crest on ninth abdominal tergum notched apically (Fig. 3I, K, L)	**2**
–	Posteromedian crest on ninth abdominal tergum tectiform apically (Fig. 3J)	***H.longiacuta* sp. nov.**
2	Pedicellus without spine on outer lateral margin (Fig. 3F–H); eighth abdominal tergum not longer than wide (Fig. 3J–L)	**3**
–	Pedicellus with a blunt spine on outer lateral margin (Fig. 3E); eighth abdominal tergum slighty longer than wide (Fig. 3I)	***H.longidentata* sp. nov.**
3	Posteromedian crest on ninth abdominal tergum larger, reaching posterior margin of anal segment (Fig, 3L)	***H.cristata* Ho, 2013**
–	Posteromedian crest on ninth abdominal tergum smaller, not reaching posterior margin of anal segment (Fig. 3K)	***H.diaoluoshanensis* Ho, 2013**

Males

**Table:** 

1	Anterior coronals lamellate (Fig. 4A, B, D); eighth abdominal tergum longer than wide	**2**
–	Anterior coronals subcylindrical (Fig. 4C); eighth abdominal tergum wider than long (Fig. 4M)	***H.diaoluoshanensis* Ho, 2013**
2	Supra-occipitals and pro-coronals present as blunt spines (Fig. 4A, D)	**3**
–	Supra-occipitals present as indistinct elevations; pro-coronals present as slender spines (Fig. 4B)	***H.longiacuta* sp. nov.**
3	Central coronal subcylindrical and slender (Fig. 4A)	***H.longidentata* sp. nov.**
–	Central coronal carinate (Fig. 4D)	***H.cristata* Ho, 2013**

**Figure 3.**
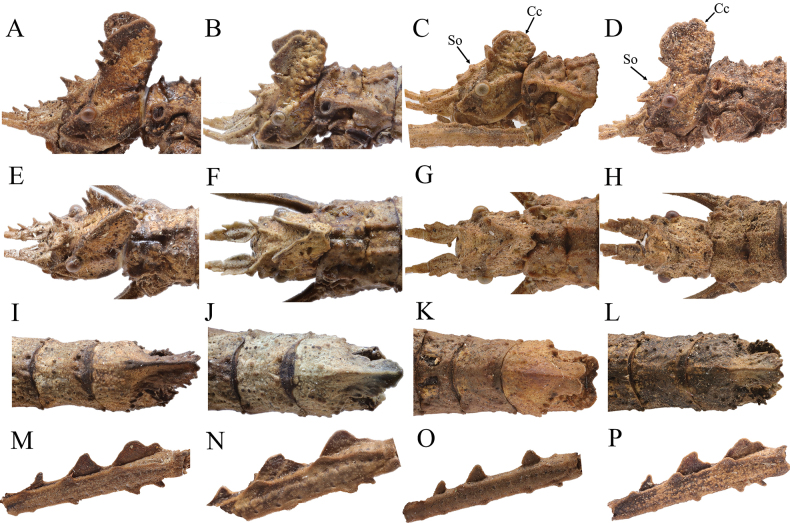
*Hainanphasma* spp., diagnostic characters of females **A–D** lateral view of head **A***H.longidentata* sp. nov. **B***H.longiacuta* sp. nov. **C***H.diaoluoshanensis* Ho, 2013 **D***H.cristata* Ho, 2013 **E–H** dorsal view of head **E***H.longidentata* sp. nov. **F***H.longiacuta* sp. nov. **G***H.diaoluoshanensis* Ho, 2013 **H***H.cristata* Ho, 2013 **I–L** dorsal view of terminalia **I***H.longidentata* sp. nov. **J***H.longiacuta* sp. nov. **K***H.diaoluoshanensis* Ho, 2013 **L***H.cristata* Ho, 2013 **M–P** lateral view of metafemur **M***H.longidentata* sp. nov. **N***H.longiacuta* sp. nov. **O***H.diaoluoshanensis* Ho, 2013 **P***H.cristata* Ho, 2013. So = supra-occipitals; Cc = central coronal. Not to scale.

**Figure 4.**
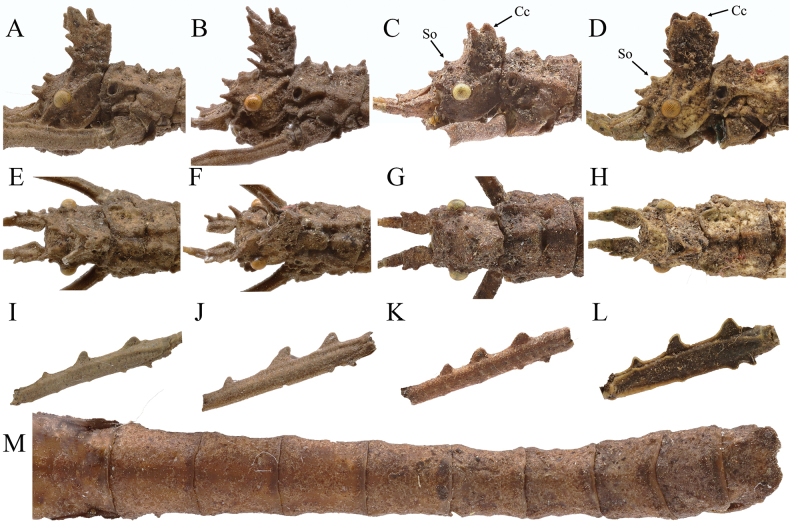
*Hainanphasma* spp., diagnostic characters of males. **A–D** lateral view of head **A***H.longidentata* sp. nov. **B***H.longiacuta* sp. nov. **C***H.diaoluoshanensis* Ho, 2013 **D***H.cristata* Ho, 2013 **E–H** dorsal view of head **E***H.longidentata* sp. nov. **F***H.longiacuta* sp. nov. **G***H.diaoluoshanensis* Ho, 2013 **H***H.cristata* Ho, 2013 **I–L** lateral view of metafemur **I***H.longidentata* sp. nov. **J***H.longiacuta* sp. nov. **K***H.diaoluoshanensis* Ho, 2013 **L***H.cristata* Ho, 2013 **M***H.diaoluoshanensis* Ho, 2013, dorsal view of abdomen. So = supra-occipitals; Cc = central coronal. Not to scale.

### ﻿ Hainanphasma longidentata sp. nov.

Taxon classificationAnimaliaPhasmidaHeteropterygidae

31A5EE28-5DD5-5C2C-A8C3-E973F7C4E51C

https://zoobank.org/B40561A6-4568-4B43-82CF-5D0D61EFDF5D

Figs 5 , 6

#### Type material.

***Holotype*.** China • ♀; Hainan Province, Lingshui County, Diaoluoshan National Nature Reserve; 19 Aug. 2023; Yifan Liu leg.; SICAHN 23011. ***Paratype*.** China • 1♂; same data as for holotype; SICAHN 23012 (all deposited in SICAU).

#### Diagnosis.

**Figure 5.**
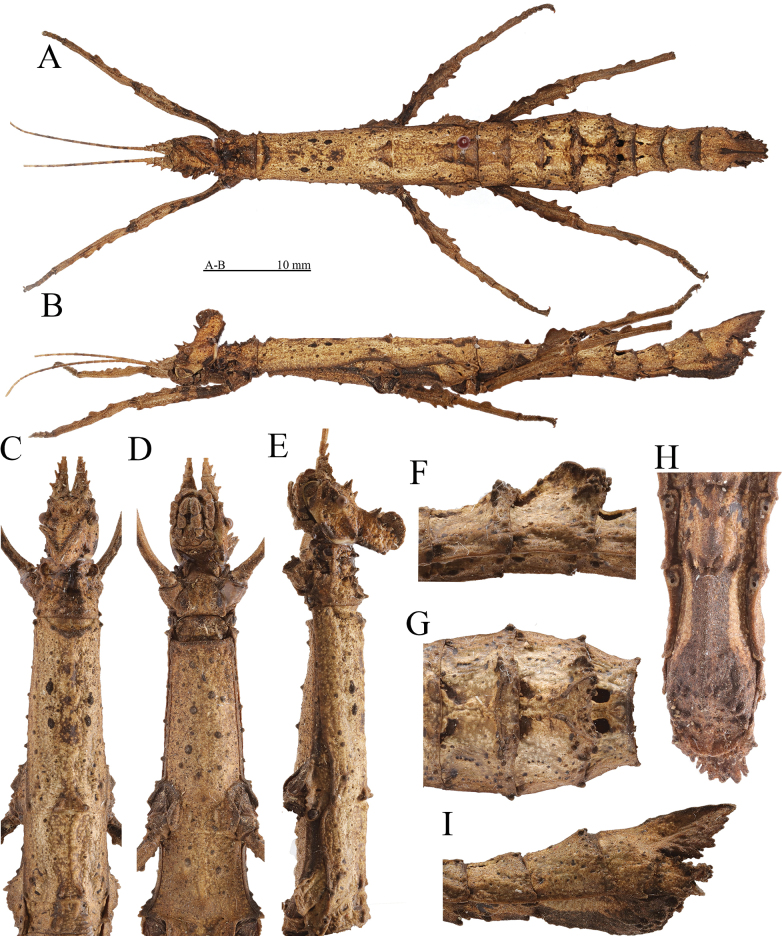
Female (HT) of *Hainanphasmalongidentata* sp. nov. **A** dorsal view of habitus **B** lateral view of habitus **C** dorsal view of head and thorax **D** ventral view of head and thorax **E** lateral view of head and thorax **F** lateral view of fourth to fifth abdominal terga **G** dorsal view of fourth to fifth abdominal terga **H** ventral view of terminalia **I** lateral view of terminalia. Scale bars: 10 mm (**A, B**).

In female, scapus with two blunt spines and pedicellus with a blunt spine on outer lateral margin (Fig. 5C); crest of head distinctly raised and elongated, with posterior margin truncate in lateral view (Fig. 5E); eighth abdominal tergum rectangular, slightly longer than wide (Fig. 5A); posteromedian crest on ninth abdominal tergum distinctly surpassing posterior margin of anal segment, with lateral margins with three to four small lamellate teeth, notched apically (Fig. 5A, I).

**Figure 6.**
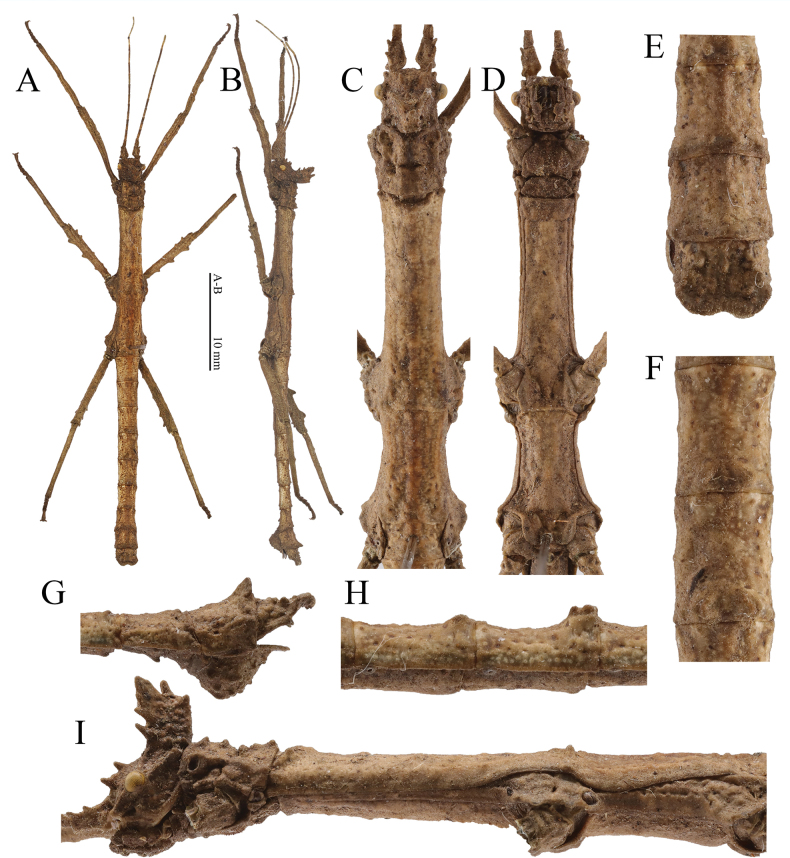
Male (paratype) of *Hainanphasmalongidentata* sp. nov. **A** dorsal view of habitus **B** lateral view of habitus **C** dorsal view of head and thorax **D** ventral view of head and thorax **E** dorsal view of terminalia **F** dorsal view of fourth to fifth abdominal terga **G** lateral view of terminalia **H** lateral view of fourth to fifth abdominal terga **I** lateral view of head and thorax. Scale bars: 10 mm (**A, B**).

In male, supra-antennals, supra-occipitals and pro-coronals present as blunt spines; anterior coronals consisting of a subtriangular lamella and a small blunt spine; central coronal slender and subcylindrical (Fig. 6I); abdominal terga with median longitudinal carina (Fig. 6A); ninth abdominal tergum with a posteromedian ridge-like structure elongated (Fig. 6G); poculum with posterior rim deeply notched posteromedially.

#### Description.

**Female** (Fig. 5).

***Head.*** Supra-antennals elongated and blunt, slightly flattened laterally and pointed outwards, about equal in length to pro-coronals, followed by two smaller supra-occipitals present as blunt spines. Supra-orbitals absent. Pro-coronals and anterior, central and posterior coronals fused basally and raised. Pro-coronals present as blunt spines. Anterior and central coronals almost continuous, merged into two carinae, strongly compressed laterally and lamellate, V-shaped in dorsal view. Posterior and lateral coronals present as small granules. Eyes dark brown, rounded. Postocular carina distinct, reaching posterior margin of head, with a conical tubercle apically. Antennae brown, darker in some segments, shorter than forelegs. Scapus strongly flattened dorsally, longer than pedicellus, with a central and subapical blunt spine on outer lateral margin; pedicellus slightly flattened dorsoventrally, shorter than third segment, with a central blunt spine on outer lateral margin; third segment cylindrical, slightly shorter than scapus.

***Thorax.*** Pronotum trapezoidal, slightly widening towards the posterior, shorter than head; anterior margin slightly concave and posterior margin nearly truncate; anterolateral angles granulose. Transverse sulcus distinct and incurved, not reaching lateral margins. Prozona with two pairs of granules along distinct longitudinal sulcus. Metazona with three pairs of granules on two indistinct longitudinal carinae, along indistinct longitudinal sulcus. Mesonotum subtrapezoidal, widening towards the posterior; median longitudinal carina indistinct, bifurcated and thickened posteriorly; anterior margin thickened and broadly concave; lateral margins with a small granule near anterior margin. Mesopleura with three or four small granules, widened above coxae; widened portion separated by an indistinct short transverse sulcus; anterior portion subtriangular, with two small granules medially on outer lateral margin; posterior portion with two small elevations anteriorly on outer lateral margin. Metanotum rectangular in outline, about 3 times as long as median segment; median longitudinal carina distinct, bifurcated posteriorly. Metapleura widened above coxae; widened portion separated by a short oblique sulcus; anterior portion with two granules medially on outer lateral margin; posterior portion with a granule medially on outer lateral margin. Mesosternum and metasternum with dispersed conical black elevations.

***Abdomen.*** Median segment to fourth terga with an x-shaped carina medially. Median segment transverse, with anterior and posterior margins nearly truncate. Second tergum parallel-sided, longer than median segment. Third to fourth terga gradually increased in width. Fourth tergum with median longitudinal carina distinct, bifurcated and lamellate posteriorly. Fifth tergum obliquely ascending, with two distinct trapezoidal tubercles posteromedially; tubercles projecting over posterior margin, covered with granules of irregular sizes; median longitudinal carina distinct, bifurcated posteriorly, with curled lamellae. Fifth to eighth terga gradually decreased in width. Eighth tergum rectangular, slightly longer than wide, with lateral margins slightly concave. Ninth tergum equal in width to anal segment, with a distinct posteromedial crest; crest elongated posteriorly, notched apically, distinctly surpassing posterior margin of anal segment; lateral margins with three or four teeth of irregular sizes. Anal segment obliquely descending and dorsally flattened, slightly narrower than ninth tergum, with two granules on oblique longitudinal carinae; dorsal surface covered with dense granules; posterolateral angles elevated and extended; posterior margin with two small granules medially. Subgenital plate boat-shaped, with distinct median carina and dense granules of irregular sizes; posterior rim dorsoventrally flattened and rounded apically, not reaching posterior margin of anal segment. Cerci flattened, hidden inside the anal segment, with apex rounded.

***Legs.*** Profemora slightly curved basally; antero-dorsal carina distinct and undulate; postero-dorsal carina indistinct, with four indistinct elevations; ventral carinae indistinct except for medio-ventral carina, with indistinct elevations. Antero-dorsal carina of meso- and metafemora with four small teeth, almost equal in size. Postero-dorsal carina of meso- and metafemora with three distinct fin-like lamellae, increasing in size towards the apex. Antero-ventral and postero-ventral carinae of meso- and metafemora with four to five teeth, two ones larger subapically. Tibiae with carinae, shorter than corresponding femora; ventral carinae unarmed. Protibiae with distinct dorsal carinae with three lamellate elevations. Meso- and metatibiae with indistinct carinae; antero-dorsal carina with a small elevation; postero-dorsal carina with a fin-like lamella medially.

**Male** (Fig. 6).

***Head.*** Supra-antennals distinct, slightly flattened laterally, blunt and slightly pointed outwards. Supra-occipitals smaller than supra-antennals, present as conical blunt spines. Pro-coronals and anterior, central and posterior coronals fused basally and distinctly raised. Pro-coronals present as blunt spines, about equal in length to supra-antennals. Anterior coronals consisting of two portions; the upper portion subtriangular and lamellate; the lower portion present as blunt spines, pointed forwards. Central coronal subcylindrical and slender, with apex rounded. Posterior and lateral coronals present as small granules. Eyes yellowish brown, rounded. Postocular carina distinct, with a small conical tubercle apically. Antennae grayish brown to dark brown, darker in some segments, shorter than forelegs. Scapus flattened dorsally, longer than pedicellus, with a central and a subapical blunt spine on outer lateral margin, the latter larger; pedicellus subcylindrical, shorter than third segment; third segment cylindrical, shorter than scapus.

***Thorax.*** Pronotum slightly widening towards the posterior, longer than head; anterior margin concave and posterior margin convex. Transverse sulcus distinct and incurved, not reaching lateral margins. Prozona distinctly elevated centrally, with two pairs of granules alone indistinct longitudinal sulcus. Metazona with two pairs of elevations and a pair of granules on two longitudinal carinae, along indistinct longitudinal sulcus. Mesonotum with indistinct median longitudinal carina, bifurcated and thickened posteriorly; anterior margin thickened and broadly concave, with three indistinct elevations; lateral margins with a row of small pits; anterolateral angles with a small granule. Mesopleura widened above coxae; widened portion separated by an indistinct transverse sulcus, with outer lateral margin undulate. Metanotum rectangular, about 2.8 times as long as median segment, with posterior margin with two elevations; median longitudinal carina distinct. Metapleura widened above coxae; widened portion separated by a short oblique sulcus; anterior portion with outer lateral margin notched medially; posterior portion subtriangular, with a small triangular lamella medially on outer lateral margin.

***Abdomen.*** Abdominal terga rough, slightly varying in length, with median longitudinal carina. Median segment with anterior margin slightly convex and posterior margin truncate. Second to third terga gradually decreasing in width, with a pair of elevations posteromedially. Second tergum rectangle, longer than wide, about 1.5 times as long as median segment. Fourth to sixth terga equal in width, with lateral margins broadly concave. Fourth and fifth terga raised posteromedially and with a pair of humps, posterior one larger. Sixth to eighth terga with a pair of granules posteromedially. Seventh tergum narrowest of abdominal terga. Eighth tergum widening towards the posterior. Ninth tergum with a ridge-like and elongated structure posteromedially. Anal segment transverse, shortest among three terminal terga, notched posteromedially; posterolateral angles rounded. Poculum more or less triangular in lateral view, with numerous granules of irregular sizes; posterior rim dorsoventrally flattened and deeply notched posteromedially; posterolateral angles rounded. Cerci small and flattened, not reaching posterior margin of the anal segment, with apex rounded.

***Legs.*** Profemora slightly curved basally; antero-dorsal carina distinct and undulate; postero-ventral carina with four indistinct elevations. Postero-dorsal carina of meso- and metafemora with three fin-like teeth, increasing in size towards the apex. Antero-dorsal carina of meso- and metafemora indistinct and unarmed. Antero-ventral and postero-ventral carinae of meso- and metafemora with two small teeth subapically, and with two indistinct elevations medially and basally. Tibiae with indistinct carinae except for protibiae, shorter than corresponding femora. Protibiae with distinct dorsal carinae with indistinct elevations. Meso- and metatibiae with carinae indistinct and unarmed.

#### Measurements (mm).

Body length: ♀56.60, ♂43.39; length of head: ♀5.22, ♂3.11; length of pronotum: ♀4.10, ♂2.85; length of mesonotum: ♀12.89, ♂9.08; length of metanotum: ♀6.55, ♂5.15; length of median segment: ♀2.13, ♂1.84; length of profemora: ♀9.98, ♂8.71; length of mesofemora: ♀8.09, ♂7.44; length of metafemora: ♀10.64, ♂9.62; length of protibiae: ♀8.32, ♂8.21; length of mesotibiae: ♀6.94, ♂6.75; length of metatibiae: ♀10.27, ♂7.96.

#### Etymology.

The name is derived from the Latin words *longi* (long) and *dentata* (toothed) and refers to the elongated posteromedian crest with dentate margins on the ninth abdominal tergum in female.

#### Distribution.

Known only from the type locality.

### ﻿ Hainanphasma longiacuta sp. nov.

Taxon classificationAnimaliaPhasmidaHeteropterygidae

EED49456-F934-55C6-A6E8-200B4014E68E

https://zoobank.org/F21F3C0D-2819-4202-A496-9B062551C527

Figs 7 , 8 , 10A–C

#### Type material.

***Holotype*.** China • ♀; Hainan Province, Ledong County, Jianfengling National Nature Reserve; 2 Aug. 2023; Yifan Liu leg.; SICAHN 23022. ***Paratype*.** China • 1♂; same data as for holotype; SICAHN 23023 (all deposited in SICAU).

#### Diagnosis.

**Figure 7.**
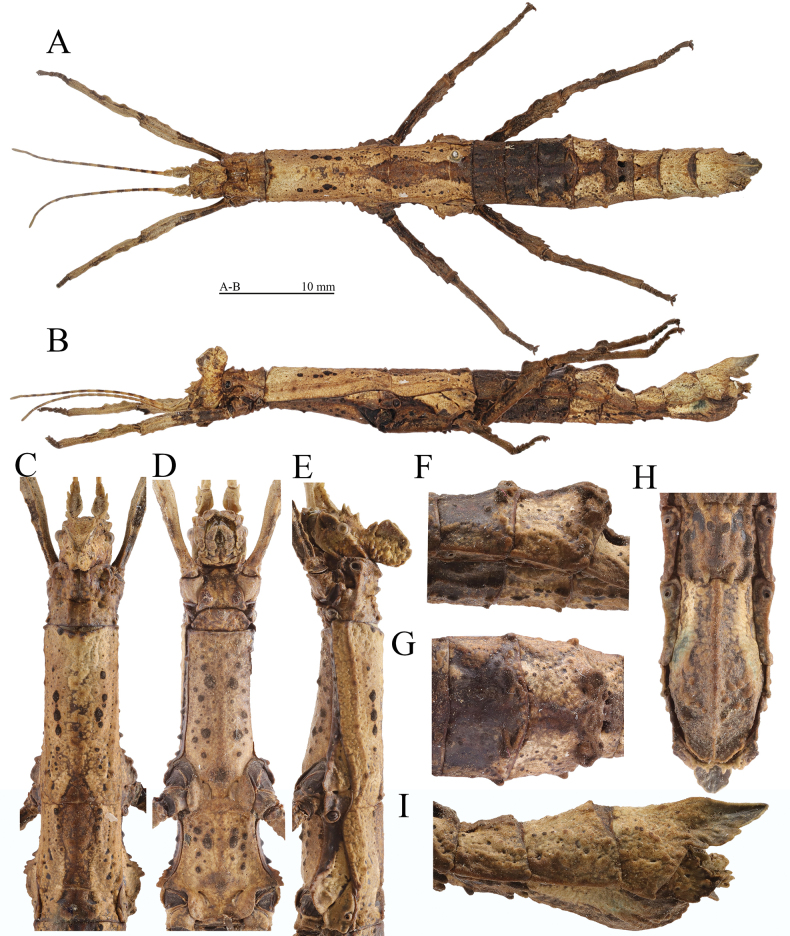
Female (HT) of *Hainanphasmalongiacuta* sp. nov. **A** dorsal view of habitus **B** lateral view of habitus **C** dorsal view of head and thorax **D** ventral view of head and thorax **E** lateral view of head and thorax **F** lateral view of fourth to fifth abdominal terga **G** dorsal view of fourth to fifth abdominal terga **H** ventral view of terminalia **I** lateral view of terminalia. Scale bars: 10 mm (**A, B**); **C–I** not to scale.

In female, scapus with a subapical blunt spine and a central indistinct elevation on outer lateral margin (Fig. 7C); pro-coronals present as subtriangular lamellae; central coronal carinate (Fig. 7E); eighth abdominal tergum wider than long (Fig. 7A); posteromedian crest on ninth abdominal tergum distinctly surpassing posterior margin of anal segment, tapering apically, with apex blunt. (Fig. 7A, H, I).

**Figure 8.**
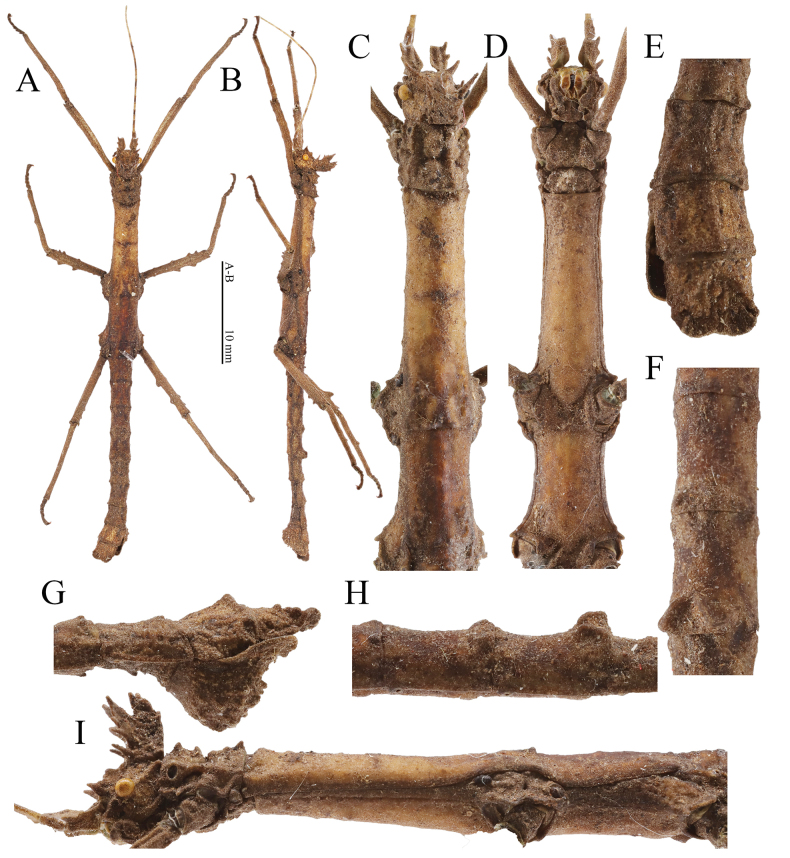
Male (paratype) of *Hainanphasmalongiacuta* sp. nov. **A** dorsal view of habitus **B** lateral view of habitus **C** dorsal view of head and thorax **D** ventral view of head and thorax **E** dorsal view of terminalia **F** dorsal view of fourth to fifth abdominal terga **G** lateral view of terminalia **H** lateral view of fourth to fifth abdominal terga **I** lateral view of head and thorax. Scale bars: 10 mm (**A, B**); **C–I** not to scale.

In male, supra-antennals distinct, present as blunt spines; supra-occipitals just present as small elevations; pro-coronals elongated and present as slender spines; anterior coronals consisting of a subtriangular lamella and a blunt spine; central coronal robust, with two granules apically (Fig. 8I); anal segment square in outline, about equal in length to ninth abdominal tergum (Fig. 8E).

#### Description.

**Female** (Fig. 7).

***Head.*** Supra-antennals slightly flattened laterally, larger than supra-occipitals; apices blunt and slightly pointed outwards. Supra-occipitals present as conical spines. Pro-coronals and anterior, central and posterior coronals fused basally and raised. Supra-orbitals absent. Pro-coronals present as subtriangular lamellae, with apices blunt, about as long as supra-antennals. Anterior coronals strongly compressed laterally and lamellate, lower than central coronal. Central coronal carinate. Posterior coronals present as small granules. Lateral coronals indistinct. Eyes brown to black brown, rounded. Postocular carina distinct, reaching posterior margin of head, with an indistinct granule apically. Antennae brown, darker in some segments, slightly shorter than forelegs. Scapus strongly flattened dorsally, longer than pedicellus, with a subapical blunt spine and a central indistinct elevation on outer lateral margin; pedicellus slightly flattened dorsoventrally, carinate laterally, shorter than third segment; third segment cylindrical, shorter than scapus.

***Thorax.*** Pronotum trapezoidal, slightly increasing in width posteriorly, about equal in length to head; anterior margin concave and posterior margin nearly truncate; lateral margins with four dispersed small granules; anterolateral angles rounded and granulose. Transverse sulcus distinct and incurved, not reaching lateral margins. Prozona with two pairs of granules along distinct longitudinal sulcus. Metazona with three pairs of small granules on two longitudinal carinae, along indistinct longitudinal sulcus. Mesonotum almost parallel-sided, with dispersed dark spots of irregular sizes; median longitudinal carina indistinct, bifurcated and thickened posteriorly; anterior margin thickened and incurved; anterolateral angles with a small granule. Mesopleura with five small granules, widened above coxae; widened portion separated by a short transverse sulcus; anterior portion subtriangular, with a small granule anteriorly and a subtriangular elevation medially on outer lateral margin; posterior portion with outer lateral margin undulate. Metanotum rectangular, about 2.5 times as long as median segment; median longitudinal carina distinct, bifurcated posteriorly. Metapleura widened above coxae; widened portion separated by a short oblique sulcus; anterior portion with consecutive three elevations medially on outer lateral margin; posterior portion with a granule medially on outer lateral margin. Mesosternum and metasternum with rounded dark elevations.

***Abdomen.*** Median segment transverse, with anterior and posterior margins nearly truncate. Second tergum parallel-sided, longer than median segment. Third and fourth terga gradually increasing in width. Fourth tergum with two pairs of oblique and short carinae posteriorly. Fifth tergum obliquely ascending, with two distinct trapezoidal tubercles posteromedially; tubercles projecting over posterior margin, covered with small granules; median longitudinal carina distinct, bifurcated and curled posteriorly. Fifth to seventh terga gradually decreased in width. Eighth tergum transverse, wider than long, equal in width to ninth tergum. Ninth tergum wider than anal segment, with a distinct posteromedian crest; crest distinctly surpassing posterior margin of anal segment, tapering apically, with apex blunt; lateral margins with one triangular lamella or three small elevations. Anal segment parallel-sided, obliquely descending, with two granules on oblique longitudinal carinae; dorsal surface covered with dense granules; posterolateral angles elevated and extended; posterior margin with two granules medially. Subgenital plate boat-shaped, with distinct median carina and dense granules of irregular sizes; posterior rim dorsoventrally flattened, notched posteromedially, not reaching posterior margin of anal segment. Cerci flattened, hidden inside the anal segment.

***Legs.*** Profemora slightly curved basally; antero-dorsal carina distinct and undulate; postero-dorsal and ventral carinae indistinct, with indistinct lamellate elevations. Antero-dorsal carina of meso- and metafemora with three to four small lamellae. Postero-dorsal carina of mesofemora with three fin-like lamellae, increasing in size towards the apex. Postero-dorsal carina of metafemora with three fin-like lamellae, median one largest. Antero-ventral and postero-ventral carinae of meso- and metafemora with four to six teeth of irregular sizes, subapical two ones larger and adjacent. Tibiae with carinae, shorter than corresponding femora; ventral carinae indistinct and unarmed. Protibiae with distinct dorsal carinae with three lamellate elevations. Meso- and metatibiae with indistinct dorsal carinae with one to two lamellate elevations anteriorly and medially.

**Male** (Fig. 8).

***Head.*** Supra-antennals distinctly elongated, blunt and spinose, slightly flattened laterally, pointed outwards. Supra-occipitals indistinct, present as small elevations. Pro-coronals and anterior, central and posterior coronals fused basally and distinctly raised. Pro-coronals distinctly elongated, present as slender spines, with apices pointed. Anterior coronals laterally compressed, consisting of a subtriangular lamella tapering apically and a spine. Central coronal robust, with two granules apically. Posterior and lateral coronals present as small granules. Eyes yellowish brown, rounded. Postocular carina indistinct, with a minute granule apically. Antennae yellowish brown to grayish brown, darker in some segments, shorter than forelegs. Scapus flattened dorsally, longer than pedicellus, with a central elevation and a subapical blunt spine on outer lateral margin; pedicellus subcylindrical, shorter than third segment, laterally carinated; third segment cylindrical, equal in length to scapus.

***Thorax.*** Pronotum rectangular, about equal in length to head; lateral margins undulate; anterior margin concave and posterior margin slightly convex. Transverse sulcus distinct and incurved, not reaching lateral margins. Prozona distinctly elevated centrally, with two pairs of granules; longitudinal sulcus absent. Metazona with two pairs of elevations and a pair of granules posteriorly on two longitudinal carinae, along indistinct longitudinal sulcus. Mesonotum slightly widening towards the posterior, with indistinct median longitudinal carina; anterior margin thickened and broadly concave; anterolateral angles with a small granule; posterior margin truncate, with two small granules medially. Mesopleura widened above coxae; widened portion separated by an indistinct transverse sulcus; anterior portion subtriangular, with a small granule anteriorly on outer lateral margin; posterior portion with outer lateral margin undulate. Metanotum approximately rectangular, about 3 times as long as median segment; median longitudinal carina distinct, with two small granules posteriorly. Metapleura widened above coxae; widened portion separated by an indistinct and oblique sulcus; anterior and posterior portion with a small granule medially on outer lateral margin.

***Abdomen.*** Abdominal terga rough, slightly varying in length. Median segment with anterior margin convex and posterior margin almost truncate. Second tergum longer than wide, slightly narrowing towards the posterior, about 1.3 times as long as median segment. Third to fifth terga equal in length, with indistinct median longitudinal carina and lateral margins broadly concave. Fourth and fifth terga thickened and with a pair of humps posteromedially, posterior one larger. Sixth to seventh terga equal in width, shorter than fifth tergum, with median longitudinal carina and lateral margins broadly concave. Eighth to ninth terga gradually increasing in width. Ninth tergum with distinct median longitudinal carina gradually ascending, present as ridge-like structure posteromedially. Anal segment square in outline, dorsoventrally flattened, notched posteromedially, equal in length to ninth tergum; posterolateral angles rounded. Poculum more or less triangular in lateral view, with numerous granules of irregular sizes; posterior rim flattened dorsoventrally and notched posteromedially; posterolateral angles rounded. Cerci small and flattened, not reaching posterior margin of the anal segment, with apex rounded.

***Legs.*** Profemora slightly curved basally; antero-dorsal carina distinct and undulate; postero-ventral carina with three indistinct elevations. Postero-dorsal carina of mesofemora with two fin-like teeth; postero-dorsal carina of metafemora with three fin-like teeth, median one largest. Antero-dorsal carina of meso- and metafemora indistinct and unarmed. Antero-ventral and postero-ventral carinae of meso- and metafemora with two small teeth subapically, and with two indistinct elevations medially and basally. Tibiae with indistinct carinae except for protibiae, shorter than corresponding femora. Protibiae with distinct dorsal carinae with an indistinct elevation medially. Meso- and metatibiae with indistinct postero-dorsal carina with an indistinct elevation medially.

**Eggs** (Fig. 10A–C). Capsule brown to dark brown, with small brownish elevations. Capsule and operculum covered with very sparse, short dark setae and densely punctulate. Micropylar plate trilobate with one anterior expansion and with two posterior expansions; anterior expansion slightly widening towards the capsule; posterior arms laterally directed and faded, not reaching ventral margin. Margin of micropylar plate elevated and widened. Micropylar cup distinct, placed near the posterior end of the micropylar plate. Operculum oval, elevated centrally; outer rim dark, with brownish elevations of irregular shape. Median line dark and indistinctly raised. Ventral margin oblique in lateral view.

#### Measurements (mm).

Body length: ♀51.89, ♂41.46; length of head: ♀4.55, ♂3.05; length of pronotum: ♀4.27, ♂2.95; length of mesonotum: ♀11.29, ♂9.24; length of metanotum: ♀5.59, ♂4.96; length of median segment: ♀2.10, ♂1.65; length of profemora: ♀8.92, ♂8.66; length of mesofemora: ♀8.67, ♂6.73; length of metafemora: ♀10.33, ♂9.28; length of protibiae: ♀7.47, ♂7.61; length of mesotibiae: ♀6.72, ♂6.17; length of metatibiae: ♀8.21, ♂7.74.

#### Etymology.

The name is derived from the Latin words *longi* (long) and *acuta* (sharpened) and refers to the posteromedian crest on ninth tergum elongated and tapering apically in female.

#### Distribution.

Known only from the type locality.

### ﻿ Hainanphasma diaoluoshanensis

Taxon classificationAnimaliaPhasmidaHeteropterygidae

Ho, 2013

574BAAE8-289C-5F44-8931-7B7DCBE95DED

Figs 9 , 10D–F

**Figure 9.**
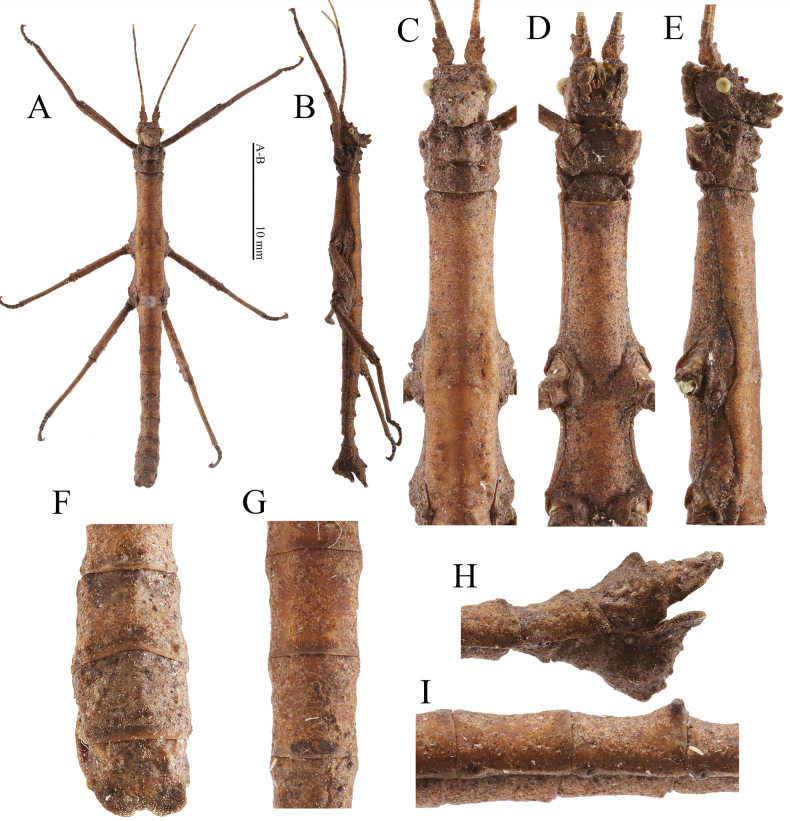
Male of *Hainanphasmadiaoluoshanensis* Ho, 2013, from China, Hainan, Lingshui, 20 Aug. 2023, Yifan Liu leg. **A** dorsal view of habitus **B** lateral view of habitus **C** dorsal view of head and thorax **D** ventral view of head and thorax **E** lateral view of head and thorax **F** dorsal view of terminalia **G** dorsal view of fourth to fifth abdominal terga **H** lateral view of terminalia **I** lateral view of fourth to fifth abdominal terga. Scale bars: 10 mm (**A, B**); **C–I** not to scale.

Hainanphasma diaoluoshanensis Ho, 2013: 203: figs 7–9, 11, 14, 16.

#### Material examined.

China • 1♀1♂; Hainan Province, Lingshui County, Diaoluoshan National Nature Reserve; 20 Aug. 2023; Yifan Liu leg.; SICAHN 23038 • 2♀; Hainan Province, Lingshui County, Diaoluoshan National Nature Reserve; 9 Jul. 2024; Yifan Liu leg.; SICAHN 24041 (all deposited in SICAU).

#### Revised diagnosis.

Smallest in size compared to other species of *Hainanphasma*.

Females can be separated from the other species by the following combination of characters: Armature of head small; supra-orbitals present; eighth abdominal tergum wider than long; posteromedian crest on ninth abdominal tergum small, not reaching posterior margin of anal segment; legs with indistinct carinae.

Males can be separated from the other species by the following combination of characters: Armature of head small; anterior coronals subcylindrical (Fig. 9E); second to seventh abdominal terga without median longitudinal carina (Fig. 9A); fifth abdominal tergum with a pair of small humps posteromedially, fourth abdominal tergum absent (Fig. 9G, I); eighth abdominal tergum trapezoidal, wider than long (Fig. 9F); posterior rim of poculum truncate posteriorly.

#### Description.

**Male** (Fig. 9). ***Head.*** Supra-antennals present as small and blunt spines, pointed outwards. Supra-occipitals indistinct, present as small granules. Pro-coronals and anterior, central and posterior coronals fused basally and raised. Pro-coronals and posterior coronals present as small elevations. Anterior coronals subcylindrical, larger than supra-antennals, with apices blunt. Central coronal robust and subtriangular, with apex rounded. Lateral coronals almost absent. Eyes yellowish brown, circular and strongly projecting hemispherically. Postocular carina indistinct, with a small granule apically. Antennae yellowish brown to darker brown, shorter than forelegs. Scapus flattened dorsally, longer than pedicellus, with two adjacent and spine-like tubercles placed before the middle on outer lateral margin; pedicellus subcylindrical, shorter than third segment; third segment cylindrical, shorter than scapus.

***Thorax.*** Pronotum rectangular, slightly longer than head; anterior margin concave and posterior margin convex; anterolateral angles rounded. Transverse sulcus distinct, not reaching lateral margins. Prozona elevated centrally, with two pairs of small conical elevations along indistinct longitudinal sulcus. Metazona with two pairs of granules on two longitudinal carinae along indistinct longitudinal sulcus. Mesonotum about rectangular, without median longitudinal carina; anterior margin thickened and broadly concave, equal in width to posterior margin; anterolateral angles rounded; posterior margin with two indistinct granules. Mesopleura widened above coxae; widened portion separated by a short transverse sulcus; anterior portion subtriangular, with a small granule medially on outer lateral margin; posterior portion with outer lateral margin truncate. Metanotum approximately rectangular, about 2.5 times as long as median segment, without median longitudinal carina. Metapleura widened above coxae; widened portion triangular, with a small granule posteriorly on outer lateral margin.

***Abdomen.*** Abdominal terga rough, slightly varying in length, with posterior margin thickened. Median segment with anterior margin distinctly convex and posterior margin slightly concave. Second to seventh terga slightly decreasing in width, without median longitudinal carina. Second tergum rectangle, longer than wide, about 1.4 times as long as median segment. Fifth tergum with a pair of small humps posteromedially; fourth tergum absent. Sixth to seventh terga with posterior margin with a pair of granules. Sixth to eighth terga gradually increasing in width. Three terminal terga transverse, wider than long. Eighth abdominal tergum trapezoidal. Ninth tergum with distinct median longitudinal carina gradually ascending, present as ridge-like structure posteromedially. Anal segment transverse, dorsoventrally flattened, notched posteromedially, shortest and narrowest among three terminal terga; posterolateral angles rounded. Poculum about triangular in lateral view, with numerous granules of irregular sizes; posterior rim flattened dorsoventrally and truncate posteriorly; posterolateral angles rounded. Cerci small and flattened, not reaching posterior margin of the anal segment, with apex rounded.

***Legs.*** Legs with carinae indistinct. Profemora slightly curved basally; antero-dorsal and postero-dorsal carinae with three indistinct elevations. Postero-dorsal carina of meso- and metafemora with three thickened and fin-like teeth, increasing in size towards the apex. Antero-dorsal carina of meso- and metafemora indistinct and unarmed. Antero-ventral and postero-ventral carinae of meso- and metafemora with two apical small elevations, posterior one larger. Tibiae unarmed, shorter than corresponding femora.

**Figure 10.**
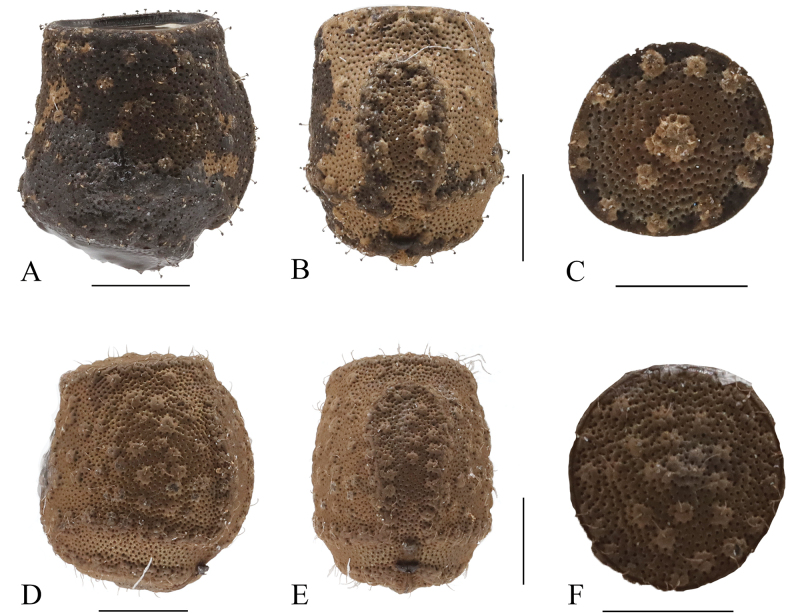
*Hainanphasma* spp., eggs. **A–C***H.longiacuta* sp. nov. **A** lateral view **B** dorsal view **C** operculum **D–F***H.diaoluoshanensis* Ho, 2013 **D** lateral view **E** dorsal view **F** operculum. Scale bars: 1 mm.

**Eggs** (Fig. 10D–F). Capsule brown, with small brownish elevations. Capsule and operculum bearing very sparse, short pale setae and densely punctulate. Micropylar plate trilobate with one anterior expansion and with two posterior expansions; anterior expansion slightly widening towards the capsule; posterior arms laterally directed, almost reaching ventral margin. Margin of micropylar plate elevated and widened. Micropylar cup distinct, placed near the posterior end of the micropylar plate. Operculum subcircular, elevated centrally, with irregularly shaped dense brownish elevations. Median line indistinctly raised. Ventral margin oblique in lateral view.

#### Redescription of female.

***Head.*** Supra-antennals present as conical spines, slightly larger than supra-occipitals. Supra-occipitals present, small and conical. Pro-coronals and anterior, central and posterior coronals fused basally and raised. Supra-orbitals and pro-coronals present as conical spines, posterior one larger. Anterior coronals slightly flattened laterally. Central coronal carinate, almost fused with anterior coronals. Posterior and lateral coronals just present as small granules. Postocular carina present, reaching posterior margin of head, with an indistinct granule apically. Antennae brown, darker in some segments, shorter than forelegs, with dense and minute brownish setae. Scapus flattened dorsally, with two adjacent and spine-like tubercles placed before the middle on outer lateral margin; pedicellus subcylindrical, shorter than third segment; third segment cylindrical, shorter than scapus.

#### Measurements (mm).

Body length: ♀44.24–46.25, ♂33.46; length of head: ♀4.15–4.35, ♂2.45; length of pronotum: ♀3.52–3.74, ♂2.72; length of mesonotum: ♀9.33–10.2, ♂7.12; length of metanotum: ♀4.70–4.98, ♂3.82; length of median segment: ♀1.87–1.91, ♂1.55; length of profemora: ♀8.01–8.43, ♂7.48; length of mesofemora: ♀7.39–7.78, ♂6.36; length of metafemora: ♀9.71–9.89, ♂8.04; length of protibiae: ♀7.09–7.35, ♂6.77; length of mesotibiae: ♀5.84–6.76, ♂5.22 length of metatibiae: ♀8.62–8.66, ♂6.49.

#### Distribution.

China, Hainan Province, Lingshui County.

### ﻿ Hainanphasma cristata

Taxon classificationAnimaliaPhasmidaHeteropterygidae

Ho, 2013

9C74BF7E-AA35-58BA-B640-7FBB23C973E0

Hainanphasma cristata Ho, 2013: 203: figs 1–6, 10, 12, 13, 15, 17, 18.

#### Material examined.

China • 1♀1♂; Hainan Province, Ledong County, Jianfengling National Nature Reserve, 28 Jul. 2023, Yifan Liu leg.; SICAHN 23052 • 2♀; Hainan Province, Ledong County, Jianfengling National Nature Reserve; 13 Jul. 2024, Yifan Liu leg.; SICAHN 24031 (all deposited in SICAU).

#### Redescription.

**Female. *Head.*** Supra-antennals flattened laterally, distinctly larger than supra-occipitals; apices blunt and slightly pointed outwards. Supra-occipitals present as conical spines. Pro-coronals and anterior, central and posterior coronals fused basally and raised. Supra-orbitals concial or invisible. Pro-coronals present as blunt spines, very close to anterior coronals. Anterior coronals compressed laterally and lamellate. Central coronal carinate. Posterior coronals present as small granules; lateral coronals distinct. Eyes brown, rounded. Postocular carina distinct, reaching posterior margin of head, with a distinct granule apically. Antennae brown, darker in some segments, slightly longer than forelegs, with dense and minute brownish setae. Scapus strongly flattened dorsally, about equal in length to the combined of pedicellus and third segments, with a subapical elongated spine and a central spine on outer lateral margin; pedicellus slightly flattened dorsoventrally, carinate laterally, shorter than third segment; third segment cylindrical, shorter than scapus.

**Male. *Head.*** Supra-antennals slightly flattened laterally, present as blunt spines, pointed outwards. Supra-occipitals indistinct, present as small granules. Pro-coronals and anterior, central and posterior coronals fused basally and raised. Pro-coronals present as blunt spines. Anterior coronals compressed laterally and lamellate, with anterior margins truncate in lateral view. Central coronal carinate. Posterior and lateral coronals present as distinct granules. Eyes yellowish brown, circular and strongly projecting hemispherically. Postocular carina distinct, with a small granule apically. Antennae yellowish brown to darker brown, with dense and minute brownish setae. Scapus strongly flattened dorsally, longer than pedicellus, with a central elevation and a subapical blunt spine on outer lateral margin; pedicellus slightly flattened dorsoventrally, shorter than third segment; third segment cylindrical, shorter than scapus.

#### Distribution.

China, Hainan province, Ledong, Baisha, Wuzhishan and Wanning counties.

#### Remarks.

This species was collected in similar location as *H.longiacuta* sp. nov., which is characterized by pedicellus carinate laterally and lacking blunt spine on outer lateral margin in female; fourth and fifth abdominal terga with a pair of humps on posterior margin in male, but differs in females with posteromedian crest of ninth abdominal tergum not surpassing posterior margin of anal segment, notched apically; pro-coronals present as blunt spines; eighth abdominal tergum about with the same length and width, differs in males with pro-coronals blunt; anterior coronals lamellate and not divided into two portions; central coronal carinate; anal segment wider than long.

## ﻿Discussion

*Hainanphasma* is closely related to *Orestes* and was originally differentiated by body characters, but according to Bresseel and Constant (2018), these generic distinctions are weak and need additional assessment, although the eggs of *Hainanphasma* are notably different from those of *Orestes*. Morphological analysis based on new materials found distinct body characteristics that differentiate *Hainanphasma* from *Orestes*, besides the obvious differences in their eggs. *Hainanphasma* exhibits distinctive arrangements of cephalic armature, with supra-occipitals appearing as blunt spines or small elevations that are not divided into anterior and posterior supra-occipitals, and supra-orbitals are either absent or indistinct. Additionally, in female *Hainanphasma*, the fifth abdominal tergum is consistently higher than the fourth, and a larger posteromedian crest is found on the ninth abdominal tergum. These characteristics are not observed in *Orestes*.

We included the shape and size of the cephalic armature as part of the diagnosis and keys, based on the idea that the number and arrangement of the cephalic armature seem to represent useful generic characters of Datamini, whereas their shape and size seem to be species-specific (Bresseel and Constant 2018, 2020). Some species of *Orestes* with less-marked morphological differences were confirmed as distinct species (Bank et al. 2021), such as *Orestesmouhotii* and *Orestesdraegeri* Bresseel & Constant, 2018, with the main differences being only by the proportionally shorter legs and the posteromedian crest on the ninth abdominal tergum in female.

*Orestesguangxiensis* was previously thought to be distributed in Hainan, but there is a lack of conclusive evidence (Chen et al. 2002; Hennemann et al. 2008). Ho (2013) suggested it should refer to a species from *Hainanphasma*. After examining numerous specimens and making collection from Hainan, in our opinion, key evidence for the distribution of *Orestes* in Hainan is lacking.

Hainan Province, geographically isolated from southern mainland China, is the largest tropical island in China. Its complex and unique habitats are rich in biodiversity, suggesting the likelihood of undiscovered species in Hainan. More material is needed to provide a more comprehensive knowledge of the position and relationship of *Hainanphasma*.

## Supplementary Material

XML Treatment for Hainanphasma

XML Treatment for Hainanphasma longidentata

XML Treatment for Hainanphasma longiacuta

XML Treatment for Hainanphasma diaoluoshanensis

XML Treatment for Hainanphasma cristata

## ﻿Additional information

### Author ORCIDs

Yi‐Fan Liu https://orcid.org/0009-0001-1649-969X

Jun-Jie Gu https://orcid.org/0000-0003-1931-4424

Hai‐Jian Wang https://orcid.org/0000-0003-4858-5813

### Data availability

All of the data that support the findings of this study are available in the main text.
